# Copeptin levels and commonly used laboratory parameters in hospitalised patients with severe hypernatraemia - the “Co-MED study”

**DOI:** 10.1186/s13054-018-1955-7

**Published:** 2018-02-09

**Authors:** Nicole Nigro, Bettina Winzeler, Isabelle Suter-Widmer, Philipp Schuetz, Birsen Arici, Martina Bally, Julie Refardt, Matthias Betz, Gani Gashi, Sandrine A. Urwyler, Lukas Burget, Claudine A. Blum, Andreas Bock, Andreas Huber, Beat Müller, Mirjam Christ-Crain

**Affiliations:** 1 Department of Endocrinology, Diabetology and Metabolism, University Hospital Basel, Petersgraben 4, 4031 Basel, Switzerland; 2 Department of Clinical Research, University Hospital Basel, Petersgraben 4, 4031 Basel, Switzerland; 3 Medical University Clinic and Divisions of Endocrinology, Diabetology and Metabolism, Kantonsspital Aarau, Aarau, Switzerland; 4 Nephrology, Dialysis & Transplantation, Kantonsspital Aarau, Aarau, Switzerland; 5 Institute of Laboratory Medicine, Kantonsspital Aarau, Aarau, Switzerland

**Keywords:** Severe hypernatraemia, Differential diagnosis, Symptoms and characteristics, Copeptin

## Abstract

**Background:**

Hypernatraemia is common in inpatients and is associated with substantial morbidity. Its differential diagnosis is challenging, and delayed treatment may have devastating consequences. The most important hormone for the regulation of water homeostasis is arginine vasopressin, and copeptin, the C-terminal portion of the precursor peptide of arginine vasopressin, might be a reliable new parameter with which to assess the underlying cause of hypernatraemia.

**Methods:**

In this prospective, multicentre, observational study conducted in two tertiary referral centres in Switzerland, 92 patients with severe hyperosmolar hypernatraemia (Na^+^ > 155 mmol/L) were included. After a standardised diagnostic evaluation, the underlying cause of hypernatraemia was identified and copeptin levels were measured.

**Results:**

The most common aetiology of hypernatraemia was dehydration (DH) (*n* = 65 [71%]), followed by salt overload (SO) (*n* = 20 [22%]), central diabetes insipidus (CDI) (*n* = 5 [5%]) and nephrogenic diabetes insipidus (NDI) (*n* = 2 [2%]). Low urine osmolality was indicative for patients with CDI and NDI (*P* < 0.01). Patients with CDI had lower copeptin levels than patients with DH or SO (both *P* < 0.01) or those with NDI. Copeptin identified CDI with an AUC of 0.99 (95% CI 0.97–1.00), and a cut-off value ≤ 4.4pmol/L showed a sensitivity of 100% and a specificity of 99% to predict CDI. Similarly, urea values were lower in CDI than in DH or SO (*P* < 0.05 and *P* < 0.01, respectively) or NDI. The AUC for diagnosing CDI was 0.98 (95% CI 0.96–1.00), and a cut-off value < 5.05 mmol/L showed high specificity and sensitivity for the diagnosis of CDI (98% and 100%, respectively). Copeptin and urea could not differentiate hypernatraemia induced by DH from that induced by SO (*P* = 0.66 and *P* = 0.30, respectively).

**Conclusions:**

Copeptin and urea reliably identify patients with CDI and are therefore helpful tools for therapeutic management in patients with severe hypernatraemia.

**Trials registration:**

ClinicalTrials.gov, NCT01456533. Registered on 20 October 2011.

**Electronic supplementary material:**

The online version of this article (10.1186/s13054-018-1955-7) contains supplementary material, which is available to authorized users.

## Background

Dysnatraemias are common in-hospital electrolyte disturbances and are related to inequalities in water homeostasis [1–3]. Mild hypernatraemia, defined as a serum sodium value > 145 mmol/L occurs in about 1–3% of hospitalised patients [4, 5], whereas more severe hypernatraemia (i.e., > 155 mmol/L) is rarer. Mortality rates in patients with hypernatraemia are clearly increased up to 37–55% [6, 7].

Mostly, hypernatraemia represents a free water deficit in relation to the body’s sodium stores and can result from a free water loss (i.e., gastrointestinal losses, osmotic diuresis and diabetes insipidus) or a gain of sodium, typically resulting from iatrogenic sodium load. The differential diagnosis of the underlying cause of hypernatraemia is based mostly first on the measurement of urine osmolality and second on the assessment of extracellular fluid volume status. Recently, a study showed insufficient urine sampling in hospitalised patients with hyponatraemia [8], which indicated low urine collection rates in hospitalised patients. To date, urine collection rates for patients with hypernatraemia have not been reported, but in clinical practice urine sampling seems to be neglected in patients with life-threatening conditions. Furthermore, the assessment of the extracellular fluid volume status in clinical practice remains challenging and can be misleading [2, 9, 10]. Several studies show that in clinical practice correction of hypernatraemia is inadequate, and delayed treatment may have morbid clinical consequences [7, 11]. Therefore, a readily available diagnostic marker for the differential diagnosis of hypernatraemia would be of major clinical importance, especially for identification of patients with central diabetes insipidus, who need prompt treatment with desmopressin (DDAVP).

The most important hormone for the regulation of water homeostasis is arginine vasopressin (AVP). The measurement of AVP is cumbersome, but copeptin [12] is produced in equimolar amounts to AVP [13], and recent data show that copeptin levels mirror AVP levels during disorders of water balance [14, 15]. Copeptin is stable in plasma and serum and can be readily determined [13, 16, 17]. In the present study, we therefore aimed to investigate whether, compared with usual biomarkers and clinical signs, copeptin measurement improves the differential diagnosis in patients with severe hypernatraemia.

## Methods

### Study design and setting

We performed a prospective observational study at the University Hospital Basel and the Medical University Clinic Aarau, Switzerland. The Ethics Committee of Basel and Aarau approved the study protocol. Informed consent was obtained from all patients or their next of kin before enrolment. From June 2011 to March 2014, 98 consecutive patients who presented either at hospital admission or during hospital care with severe hypernatraemia, defined as serum sodium > 155 mmol/L, were included.

### Clinical variables and management of participants during the study

At study inclusion, the following data were prospectively collected: vital signs; co-morbidities; current medications; symptoms related to hypernatraemia; and laboratory parameters such as plasma osmolality, urea, uric acid and, where available, complete urine analysis. The attending physician, who was not involved in the study protocol, treated patients during hospitalisation with either glucose infusion, free water load, stopping of the iatrogenic cause, or DDAVP.

While hospitalised, patients’ serum sodium levels were measured at least daily until normalisation of sodium levels. Other laboratory measurements, such as creatinine, potassium, uric acid and urea, were performed regularly according to the treating physician. Acute kidney injury and chronic kidney disease were diagnosed according to current Kidney Disease: Improving Global Outcomes guidelines [18, 19], and the estimated glomerular filtration rate was calculated with the Chronic Kidney Disease Epidemiology Collaboration formula. During hospitalisation, every change in drugs and therapeutic management, including therapy success, was carefully monitored. Additionally, a 24-h fluid balance, including oral intake of free water, parenteral nutrition, all intravenous infusions and, where possible, urine output, was obtained daily until serum sodium levels were restored. A board-certified endocrinologist assessed volaemic status by complete clinical examination. Special attention was paid to the visual assessment of the external jugular venous pressure, presence of oedema or ascites, weight course during hospitalisation, humidity of mucous membranes and skin turgor. The final diagnosis of the underlying cause of hypernatraemia was made retrospectively by three independent investigators blinded to copeptin levels and was based on a complete chart review, including prospectively collected laboratory parameters at study inclusion and during hospitalisation (i.e., plasma osmolality, urea, uric acid, total protein, potassium, serum osmolality, creatinine, albumin and glucose), urine parameters where available (i.e., complete urine analysis), 24-h fluid balance and the entire inpatient course during hospitalisation, including treatment response to all interventions. For the adjudicated differential diagnosis, a pre-defined algorithm with four etiologic classifications was used (see Table 1 and Additional file 1). In case of discordance, patient charts were mutually discussed until a final agreement was reached.

**Table 1:** Differential diagnosis of hypernatraemia

Category	Example	Treatment
Dehydration	Extrarenal water loss (i.e., vomiting, diarrhoea, dermal losses [fever], long recumbency) Renal losses (i.e., glycosuria, urea diuresis [steroids])	Increase free water load
		Glucose 5% intravenously
		Correct other electrolytes (i.e., potassium)
Central diabetes insipidus	Central diabetes insipidus	Treat with DDAVP
Nephrogenic diabetes insipidus	Nephrogenic diabetes insipidus	Stop cause if possible
		Adequate oral fluid intake
Salt overload	Iatrogenic sodium overload (i.e., uncontrolled saline infusion, tube feedings)	Stop iatrogenic action
		Increase free water load
		Glucose 5% intravenously

*DDAVP* Desmopressin

### Blood sampling

Blood samples were collected directly on admission at the emergency department and stored at −70 °C. Measurement of copeptin levels was done in a batch analysis with a commercial sandwich immunoluminometric assay (B∙R∙A∙H∙M∙S CT-proAVP LIA; B∙R∙A∙H∙M∙S GmbH, Hennigsdorf/Berlin, Germany) as described in detail elsewhere [17]. The lower detection limit of the copeptin assay was 0.4 pmol/L, and the functional assay sensitivity was < 1 pmol/L. In healthy volunteers, median copeptin plasma concentrations are reported to be 4.2 (IQR 1.0–13.8) pmol/L [17]. Copeptin measurement is simple, and results can be obtained in < 1 h [20].

### Objectives

The primary objective of this study was to evaluate the diagnostic value of copeptin in the differential diagnosis of patients with severe hypernatraemia. Secondary objectives were the comparison of other laboratory markers in the differential diagnosis and the assessment of symptoms and clinical characteristics of patients with severe hypernatraemia.

### Statistical analysis

Discrete variables are expressed as frequency (percent), and continuous variables are expressed as median (IQR). Comparisons between groups were made using the chi-square test, the Mann-Whitney *U* test and the Kruskal-Wallis test as appropriate. In cases of a significant result, we used Dunn’s post hoc test for multiple testing to identify the specific group differences. Owing to the small number of patients with nephrogenic diabetes insipidus (*n* = 2), no statistical analyses were performed with this patient group. To quantify the accuracy of copeptin and other studied variables in predicting differential diagnoses of severe hypernatraemia, we performed ROC curve analysis, and we report AUC values and their 95% CIs. Analyses were performed using Prism version 6 software (GraphPad Software, La Jolla, CA, USA). *P* values < 0.05 were considered to indicate statistical significance.

## Results

### Baseline characteristics and symptoms

A total of 98 patients with severe hypernatraemia at admission were recruited. Six patients were excluded for missing values of copeptin (*n* = 4) or withdrawal of informed consent (*n* = 2). Therefore, the final analysis included 92 patients (93.8%). Table 2 provides baseline characteristics of the study population. At study inclusion, the median subject age (*n* = 92) was 76 (IQR 64–81) years, and 41 patients (45%) were female. The median serum sodium value at study inclusion was 159 (IQR 157–161) mmol/L. Vital signs on admission revealed a median systolic blood pressure of 130 (IQR 116–143) mmHg, a median diastolic blood pressure of 68 (IQR 56–80) mmHg, a median body temperature of 37.7 °C (IQR 36.9–38.2 °C) and a median Glasgow Coma Scale score of 13 (IQR 9–14).

**Table 2:** Baseline characteristics

Characteristics	All patients (*n* = 92)	Salt overload (*n* = 20)	Dehydration (*n* = 65)	Central diabetes insipidus (*n* = 5)	Nephrogenic diabetes insipidus (*n* = 2)^a^
Percentage of total cohort	100	22	71	5	2
Median (IQR) age, years	76 (64–81)	69 (63–77)	78 (67–82)	66 (54–70)	64
Female sex, *n* (%)	41 (45)	8 (40)	29 (45)	4 (80)	0 (0)
Hypernatraemia development during hospitalisation, *n* (%)	58 (63)	20 (100)	35 (54)	1 (20)	2 (100)
SAPS II score^b^	49 (41–59)	45 (38–57)	51 (42–59)	31 (20–58)	60
Median [IQR] laboratory variables					
Plasma sodium, mmol*/*L	159 (157–161)	157 (156–160)	160 (158–161)	158 (157–159)	157
Copeptin, pmol*/*L	51.76 (31.71–78.71)	54.95 (35.01–71.70)	53.19 (34.39–85.95)	3.39 (1.99–3.90)	77.75
Plasma osmolality, mmol*/*kg	347.5 (331–371.8)	361.5 (342.2–366.5)	349 (331–382)	346 (340–351)	337
Urine osmolality, mmol*/*L	510 (413–647)^c^	510 (462–819)	546 (463–647)	249 (216.3–280.8)	284.5
Urine sodium, mmol*/*L	60 (28–96.5)	78 (61–107)	49.6 (28.3–102.4)	33 (31–34)	77
Creatinine, μmol*/*L	121 (82–177.3)	129.5 (91–188)	119.5 (84.5–173.5)	67.5 (61.8–81.0)	317
eGFR, ml/min/1.73 m^2^	45.2 (25.8–66.9)	45.9 (25.2–60.7)	45.0 (26.4–66.6)	97.1 (48.6–110.5)	17.0
Urea, mmol*/*L	18.2 (12.5–31.6)	19.8 (15.8–34.3)	18.1 (12.8–30.6)	4 (3.1–3.7)	15.3
Uric acid, μmol*/*L	426.5 (306.5–634.8)	403 (323.5–544.5)	472 (304.8–769.5)	282 (263–296.5)	485
Median (IQR) vital signs					
Systolic blood pressure, mmHg	130 (116–143)	130 (120–143)	129 (114–143)	129 (117–138)	112
Diastolic blood pressure, mmHg	68 (56–80)	65 (51–80)	70 (57–80)	64 (53–78)	58
Heart rate, beats/minute	90 (80–104)	88 (75–91)	90 (80–109)	88 (77–110)	104
Temperature, °C	37.7 (36.9–38.2)	37.5 (37.0–38.0)	37.8 (36.9–38.2)	37.8 (37.7–38.0)	37.8
Glasgow Coma Scale score	13 (9–14)	13 (10–14)	13 (9–14)	14 (13–14)	6
Volaemic status, *n* (%)					
Hypovolaemic	63 (68)	3 (15)	55 (85)	4 (80)	1 (50)
Euvolaemic	15 (16)	5 (25)	8 (12)	1 (20)	1 (50)
Hypervolaemic	14 (15)	12 (60)	2 (3)	0 (0)	0 (0)

*Abbreviations: SAPS II* Simplified Acute Physiology Score II, *eGFR* Estimated glomerular filtration rate calculated with Chronic Kidney Disease Epidemiology Collaboration formula

^a^Only median values are reported

^b^Calculated for 81 (88%) patients

^c^Measured in total in 41% before treatment initiation (salt overload, *n* = 7; dehydration-induced, *n* = 26; central diabetes insipidus, *n* = 3; nephrogenic diabetes insipidus, *n* = 2)

Sixty-six patients (72%) were hospitalised at the general medicine ward, 15 patients (16%) at the geriatric ward and 11 patients (12%) at the general surgery ward. In total, 50 patients (54%) were hospitalised at the intensive care unit (ICU), and the mortality rate was 30.4% (*n* = 28). Altogether, we had complete data to calculate the Simplified Acute Physiologic Score II (SAPS II) for 81 patients (88%). The median SAPS II scores were similar in patients with salt overload (*n* = 18; median score 45, IQR 38–57), dehydration-induced hypernatraemia (*n* = 56; median score 51, IQR 42–59) and nephrogenic diabetes insipidus (*n* = 2; median score 60). Patients with central diabetes insipidus (*n* = 5) tended to have a lower median SAPS II score (31, IQR 20–58); however, this difference was not statistically significant (*P* = 0.14).

Until discharge from the hospital, 70 patients (76.0%) achieved a serum sodium value < 145 mmol/L, and the time to sodium value normalisation was 5 (IQR 3–7) days. Fifteen patients (71%) died before reaching serum sodium levels < 145 mmol/L, and six patients (29%) were discharged from hospital before achieving a normal sodium value (median serum sodium value 148 mmol/L, IQR 147–152).

Of the 92 patients with severe hypernatraemia, 20 (22%) had hypernatraemia due to salt overload, 65 (71%) had hypernatraemia due to dehydration, 5 patients (5%) had central diabetes insipidus and 2 patients (2%) had nephrogenic diabetes insipidus. Salt overload during hospitalisation was due to uncontrolled saline infusion in 16 patients (80%) and due to tube feeding in 4 patients (20%). Patients with dehydration-induced hypernatraemia had mostly dermal losses due to fever and sweating (*n* = 37 [57%]), followed by long recumbency at home (*n* = 16 [25%]), gastrointestinal losses (*n* = 6 [9%]) and renal losses (*n* = 6 [9%]).

Overall, hypernatraemia was present at hospital admission in 34 patients (37%) and developed during hospitalisation in 58 patients (63%). All patients with hypernatraemia due to salt overload (*n* = 20 [100%]), 35 patients (53%) with dehydration-induced hypernatraemia, both patients with nephrogenic diabetes insipidus (100%) and one patient (25%) with central diabetes insipidus developed hypernatraemia during hospitalisation.

Patients with hypernatraemia had a wide spectrum of non-specific symptoms. The most common symptoms were generalised weakness (*n* = 52 [57%]) and fatigue (*n* = 51 [55%]), disturbed gait (*n* = 19 [21%]) and recurrent (*n* = 11 [12%]) or acute falls (*n* = 13 [14%]). Fractures at time of hospitalisation or until discharge were reported for three patients (3%). Only 25 patients (27%) reported a thirst sensation at study inclusion. Seventy-one patients (77%) were disoriented at least in one quality (i.e., to the person, to location, to time or to situation). Table 3 shows the distribution of the recorded symptoms.

**Table 3:** Symptoms and co-morbidities

Characteristics	All patients (*n* = 92)	Salt overload (*n* = 20)	Dehydration (*n* = 65)	Central diabetes insipidus (*n* = 5)	Nephrogenic diabetes insipidus (*n* = 2)
Symptoms *n* (%)					
ᅟFatigue	51 (55)	10 (50)	37 (57)	4 (80)	0 (0)
Weakness	52 (57)	10 (50)	40 (62)	2 (40)	0 (0)
Disorientation	71 (77)	12 (60)	53 (82)	4 (80)	2 (100)
Disturbed gait	19 (21)	2 (10)	17 (26)	0 (0)	0 (0)
Fall	24 (26)	2 (10)	22 (34)	0 (0)	0 (0)
Headache	7 (8)	2 (10)	3 (5)	2 (40)	0 (0)
Nausea	6 (7)	1 (5)	3 (5)	1 (20)	1 (50)
Sensation of thirst	25 (27)	4 (20)	18 (28)	3 (60)	0 (0)
Co-morbidities,^a^ *n* (%)					
Hypertension	61 (66)	15 (75)	42 (65)	3 (60)	1 (50)
CNS diseases	53 (58)	8 (40)	41 (63)	3 (60)	1 (50)
Previous dysnatraemia	27 (29)	6 (30)	18 (28)	2 (40)	1 (50)
Congestive heart failure	28 (30)	7 (35)	20 (31)	1 (20)	0 (0)
Acute kidney injury	43 (46)	11 (55)	29 (45)	1 (20)	2 (100)
Chronic kidney disease	63 (68)	15 (75)	44 (68)	2 (40)	2 (100)
Pulmonary diseases	49 (53)	10 (50)	36 (55)	2 (40)	1 (50)
Medication,^a^ *n* (%)					
Loop diuretics	38 (41)	10 (50)	28 (43)	0 (0)	0 (0)
Thiazide diuretics	11 (12)	3 (15)	8 (12)	0 (0)	0 (0)
Potassium sparing diuretics	10 (11)	3 (15)	7 (11)	0 (0)	0 (0)
Opioids	21 (23)	4 (20)	16 (25)	0 (0)	1 (50)
Neuroleptics	15 (16%)	4 (20)	10 (15)	0 (0)	1 (50)
SSRI	6 (7)	2 (10)	3 (5)	0 (0)	1 (50)
NSAR	10 (11)	2 (10)	7 (11)	0 (0)	1 (50)

*Abbreviations: CNS* Central nervous system, *NSAR* Non-steroidal anti-rheumatic, *SSRI* Selective serotonin reuptake inhibitor

^a^Based on medical charts, patient report or both

The most common co-morbidities were central nervous system diseases (*n* = 53 [58%]), hypertension (*n* = 61 [66%]) and chronic kidney disease (*n* = 63 [68%]). In total, 43 patients (46%) had an acute kidney injury at study inclusion. Furthermore, 28 patients (30%) had congestive heart failure, and 49 patients (53%) had pulmonary diseases such as acute pneumonia, chronic obstructive pulmonary disease or pulmonary tumour. Twenty-seven patients (29%) had experienced hyper- or hyponatraemia in the past.

Most patients had undergone numerous drug treatments before developing hypernatraemia (either at home or in hospital). Thirty-eight patients (41%) were receiving a medication with loop diuretics, 11 patients (12%) were treated with thiazide diuretics and 10 patients (11%) were treated with potassium-sparing diuretics. Other frequently used medications were opioids (*n* = 21 [23%]) and neuroleptics (*n* = 15 [16%]). Co-morbidities and current medications of the study population are listed in Table 3.

### Differential diagnosis of hypernatraemia

**Fig. 1:**
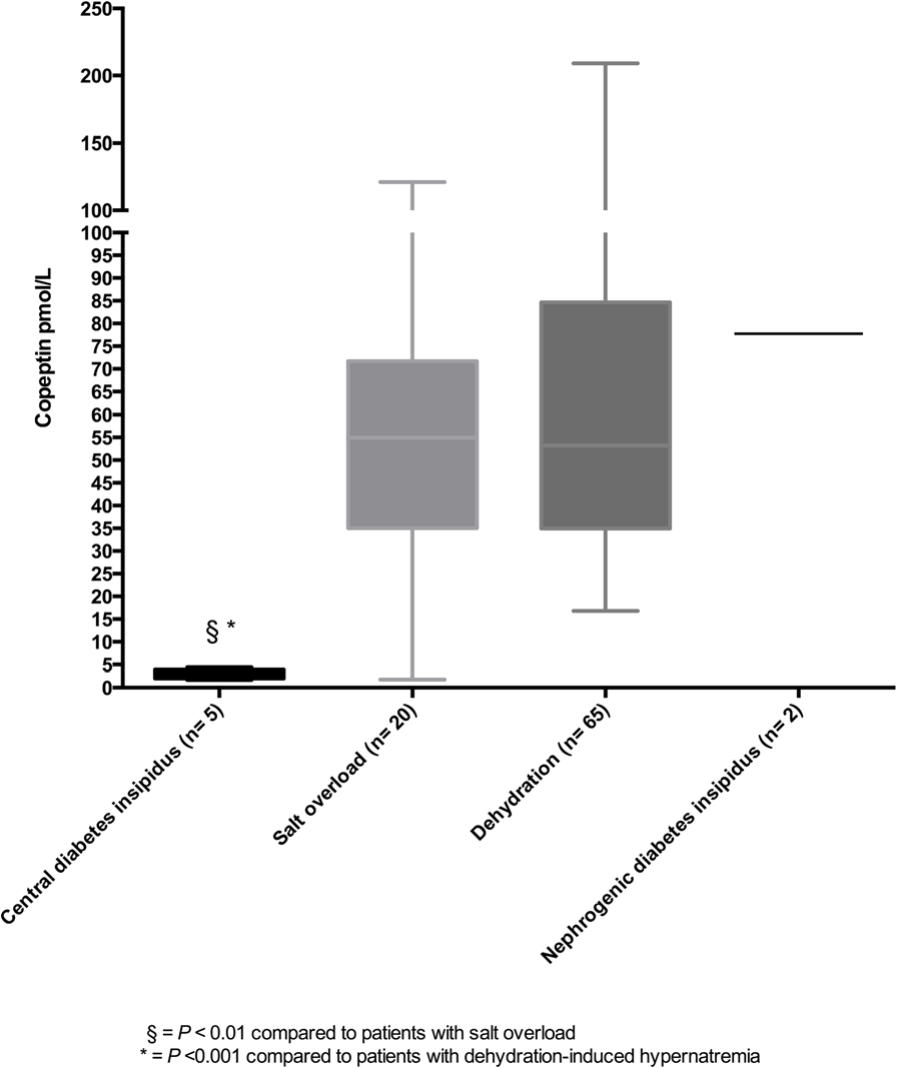
Copeptin levels in the differential diagnosis of hypernatraemia

Median copeptin levels were different in patients with central diabetes insipidus (3.39 pmol/L, IQR 1.99–3.90), salt overload (53.19 pmol/L, IQR 34.39–85.95) and dehydration-induced hypernatraemia (54.95 pmol/L, IQR 35.01–71.70) (*P* = 0.001). In a post hoc group comparison, copeptin levels in patients with central diabetes insipidus were significantly lower than in patients with salt overload or dehydration-induced hypernatraemia (*P* < 0.01 and *P* < 0.001, respectively). Copeptin levels did not differ between patients with dehydration-induced hypernatraemia and salt overload (*P* = 0.64). Patients with nephrogenic diabetes insipidus had the highest median copeptin levels (77.75 pmol/L) (*see* Fig. 1).

Copeptin levels provided good diagnostic utility for identifying patients with central diabetes insipidus compared with patients with salt overload, dehydration-induced hypernatraemia or nephrogenic diabetes insipidus, with an AUC of 0.99 (95% CI 0.97–1.00). Choosing a cut-off value of < 4.4 pmol/L, copeptin levels had a sensitivity of 100% and a specificity of 99% for diagnosing central diabetes insipidus.

The AUC to predict dehydration-induced hypernatraemia compared with salt overload by evaluating volaemic status was 0.88 (95% CI 0.78–0.98). Furthermore, volaemic status was able to predict hypernatraemia due to salt overload compared with central diabetes insipidus (AUC 0.89, 95% CI 0.75–1.00). However, volaemic status was not able to differentiate between dehydration-induced hypernatraemia compared with central diabetes insipidus (AUC 0.52, 95% CI 0.25–0.79).

**Fig. 2:**
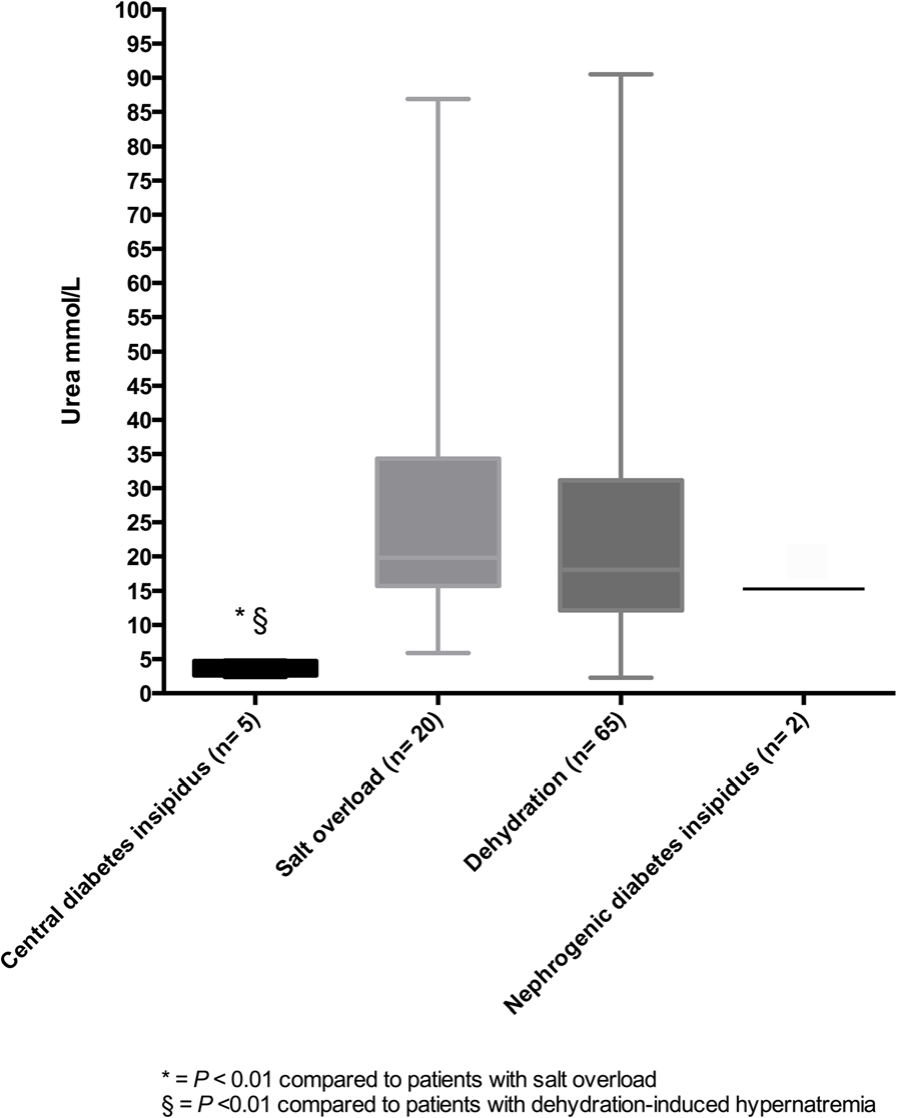
Urea levels in the differential diagnosis of hypernatraemia

Median urea values were different in patients with central diabetes insipidus, salt overload and dehydration-induced hypernatraemia (*P* = 0.003). In a post hoc comparison, median urea values in patients with central diabetes insipidus (4 mmol/L, IQR 3.1–3.7) were lower than in patients with salt overload (19.8 mmol/L, IQR 15.8–34.3) or dehydration-induced hypernatraemia (18.1 mmol/L, 12.2–30.1) (*P* < 0.01 and *P* < 0.01, respectively). Of note, urea values were similar in patients with dehydration-induced hypernatraemia and salt overload (*P* = 0.27). Patients with nephrogenic diabetes insipidus showed high median urea levels (15.3 mmol/L) (*see* Fig. 2). The AUC for urea levels in diagnosing central diabetes insipidus was 0.98 (95% CI 0.96–1.0). A cut-off value of < 5.05 mmol/L showed a specificity of 98% and a sensitivity of 100% for the diagnosis of central diabetes insipidus. When only patients hospitalised at the ICU (*n* = 50) were included, the results were similar to those for the whole patient cohort (data not shown).

Overall, urine samples collected before treatment initiation were available only for 3 patients with central diabetes insipidus, 2 patients with nephrogenic diabetes insipidus, 7 patients with salt overload and 26 patients with dehydration-induced hypernatraemia. Taken together, patients with central and nephrogenic diabetes insipidus had a lower urine osmolality (284 mmol/kg, IQR 209–306) than patients with dehydration-induced hypernatraemia (546 mmol/kg, IQR 463–647) or salt overload (510 mmol/kg, IQR 462–819) (*P* = 0.006). A urine osmolality < 329 mOsm/kg revealed a sensitivity and specificity of 100% for identifying patients with either central or nephrogenic diabetes insipidus. We found no differences between the various differential diagnoses for uric acid (*P* = 0.1) and urine sodium values (*P* = 0.47).

## Discussion

To our knowledge, this is the largest study involving prospective evaluation of symptoms and characteristics of patients with severe hypernatraemia and assessment of the value of copeptin and other laboratory parameters in the differential diagnosis of severe hypernatraemia. Our data reveal five key findings. First, patients with severe hypernatraemia had a variety of non-specific symptoms, and notably, only a few patients reported thirst. Furthermore, most patients had several co-morbidities and were treated with numerous drugs promoting the development of hypernatraemia. Second, urine osmolality could identify patients with either central or nephrogenic diabetes insipidus, but it did not differ between these two differential diagnoses. Third, copeptin levels were lowest in patients with central diabetes insipidus-induced hypernatraemia with a cut-off of 4.4 pmol/L, thus identifying patients with central diabetes insipidus with a very high specificity and sensitivity. Furthermore, low urine osmolality and high copeptin levels were diagnostic for nephrogenic diabetes insipidus. Fourth, low urea levels were found in patients with central diabetes insipidus, and levels < 5.05 mmol/L were equally specific and sensitive for the diagnosis of central diabetes insipidus. Fifth, no differentiation was possible between patients with dehydration-induced hypernatraemia and salt overload with both markers.

Our cohort is comparable to other cohorts described in the literature, including mostly elderly, general medical and typical ICU patients [11, 21, 22]. Symptoms were diffuse, and the most common recorded signs were neurological symptoms (i.e., somnolence, disorientation and falls), which is in line with a previously published retrospective study describing patients with severe hypernatraemia [11]. Interestingly, only one-fourth of our patients reported thirst sensation. One reason for this might be the high number of neurological symptoms and co-morbidities, reflecting the difficulty for patients to report thirst. However, absence of thirst is a well-known phenomenon in elderly patients [23, 24], which might have had a high impact on developing hypernatraemia in this elderly population. Furthermore, with advanced age, renal volume and the number of functioning nephrons decrease progressively, and the capacity to concentrate urine diminishes [25, 26]. Additionally, the response of renal tubules to AVP might be impaired [27]. These changes in the aging kidney can lead to reduced water retention and sodium excretion capacity, promoting the development of hypernatraemia in elderly patients.

Recently, Palevsky et al. showed that factors responsible for the development of severe hypernatraemia in non-ICU patients are increased insensible and enteral losses and urinary concentration defects, in addition to inadequate fluid management [4]. Similarly, in another study, ICU patients developed hypernatraemia because of the underlying disease or renal water loss and, most importantly, incorrect or inefficient treatment [2]. In our study, patients with hypernatraemia were frail and had various underlying pathologies promoting the development of hypernatraemia, such as heart failure, chronic kidney disease, sepsis, neurologic impairment and multiple medications. Interestingly, 63% of our patients developed hypernatraemia during hospitalisation, which is in line with the observations of Palevsky et al. [4] and Hoorn et al. [2]. These results reflect the importance of rapidly identifying the correct differential diagnosis of hypernatraemia and initiating the appropriate therapy.

Unfortunately, in clinical practice the correct differential diagnosis of hypernatraemia, which is usually based on urine osmolality and volaemic status, might be difficult, and delayed treatment may have devastating clinical consequences, leading to higher mortality rates [2, 7, 9–11, 22]. In our study, urine osmolality was able to identify patients with either central or nephrogenic diabetes insipidus. Nevertheless, urine sampling was not performed routinely in all patients, and only 41% had complete urine samples before treatment initiation. This low number reflects the general lack of urine sampling in clinical practice in patients with dysnatraemia. A large retrospective study assessing the diagnostic and therapeutic management of hyponatraemic patients showed considerable deficiencies in urine testing, confirming the results of previous small studies [8]. To our knowledge, to date, performance of urine collection in patients with hypernatraemia has not been assessed in a larger cohort, but according to our results, urine collection remains challenging in the hospital setting. Nevertheless, our results indicate that urine osmolality, which is inexpensive and usually routinely available, is a reliable marker for diagnosing either central or nephrogenic diabetes insipidus and should therefore be measured systematically in all patients with electrolyte disorders.

In a second step, differential diagnosis of hypernatraemia is usually based on volaemic status. The assessment of volume status is known to be challenging, even when performed by experienced clinicians [28]. In our study, volaemic status was able to differentiate between dehydration-induced hypernatraemia and hypernatraemia due to salt overload. This suggests that the assessment of volume status, if correctly and careful performed, adds some valuable information in the differential diagnosis of hypernatraemia. However, for differentiation between dehydration-induced hypernatraemia and central diabetes insipidus, volaemic status was not reliable.

Copeptin levels reliably identified patients with central diabetes insipidus. Low copeptin levels have already been shown to indicate central diabetes insipidus in ambulatory patients presenting with polyuria polydipsia syndrome [29] and in patients after pituitary surgery [30]. We confirm that in our cohort of hospitalised, mostly ICU patients with high sodium levels, a low copeptin level identified patients with central diabetes insipidus with high sensitivity and specificity. The cut-off level of copeptin was 4.4 pmol/L, whereas in ambulatory patients with central diabetes insipidus and patients with post-surgical central diabetes insipidus copeptin levels < 2.6 pmol/L and < 2.9 pmol/L, respectively, diagnosed diabetes insipidus [29, 30]. It is well known that copeptin is a marker for disease severity and accurately mirrors sepsis, ischaemic stroke or heart failure severity [12, 31, 32]. Therefore, on one hand, a mild stress-induced copeptin elevation could explain the higher copeptin levels in our study population with > 50% hospitalised at the ICU. On the other hand, our patients might have had only a partial AVP secretion insufficiency. This is in line with previously published results that showed an optimal copeptin cut-off level of 4.9 pmol/L for the differential diagnosis of partial central diabetes insipidus and primary polydipsia after water deprivation [33].

Our fourth observation was that low urea values were highly specific and sensitive for diagnosing central diabetes insipidus. This finding is in accordance with the results of a small retrospective study that showed low urea values in patients with central diabetes and dehydration (median serum sodium 155 mmol/L) compared with patients with dehydration not due to central diabetes insipidus [34]. The authors in that study concluded that this phenomenon is due to the net reabsorption of urea, which is dependent on the renal action of AVP. Similarly, in vitro experiments in rats and humans showed that in the inner medullary collecting tubule, urea permeability rises in response to AVP [35, 36]. Thus, urea levels might reflect AVP action in the nephrons and may differentiate patients with diabetes insipidus from dehydrated states. However, it is well known that urea levels increase with progressive decline in kidney function, and in our study population, acute as well as chronic renal impairment was more pronounced in patients with salt overload or dehydration than in our patients with central diabetes insipidus. Thus, our findings might be biased by this fact, and more studies are warranted to verify our results.

The following limitations of our study must be taken into account. First, aetiological classification of hypernatraemia is difficult. Therefore, despite careful assessment of the patients and complete chart review, it is possible that some patients were misdiagnosed. Second, although we sought to sample urine in all patients as soon as hypernatraemia was diagnosed, complete urine analysis was performed in only 41% before initiation of treatment for hypernatraemia. However, this poor collection rate reflects clinical reality, where urine collection seems to be secondary in patients with severe, often life-threatening co-morbidities, and it might have been complicated by oliguria or incontinence in our patients.

Third, copeptin levels were helpful only for the differential diagnosis of a small group of patients with central diabetes insipidus. Additionally, we had only two patients with renal diabetes insipidus, and owing to this low number, performance of statistical analysis was limited. However, previous studies showed that patients with renal diabetes insipidus have higher copeptin levels than patients with central diabetes insipidus [33], which is in line with our observation of clearly higher copeptin levels in nephrogenic diabetes insipidus than in central diabetes insipidus. Furthermore, we included patients with severe hypernatraemia, but moderate hypernatraemia is a more common problem in clinical practice, and future studies should be done to evaluate the use of copeptin in this patient population. Fourth, assessment of symptoms and their relationship to hypernatraemia is challenging, and despite careful clinical assessment including standardised bedside interview, it is possible that some patients’ symptoms were not fully recorded. The major strength of our study is that it is to date largest prospective study including evaluation of patients with severe hypernatraemia.

## Conclusions

Patients hospitalised with severe hypernatraemia are usually elderly and frail with several co-morbidities and show a variety of different, mostly neurological symptoms. Our results indicated that copeptin as well as urea levels identify patients with central diabetes insipidus with high specificity and sensitivity among patients with severe hypernatraemia, but both markers are not helpful in the differential diagnosis between dehydration-induced hypernatraemia and salt overload. Urine osmolality reliably identified patients with central or nephrogenic diabetes insipidus, but overall urine collection before treatment initiation was poor.

## Additional file

Additional file 1: Clinical diagnostic algorithm for the differential diagnosis of hypernatremia. (TIFF 143 kb)

## Abbreviations

AVP: Arginine vasopressin
CDI: Central diabetes insipidus
CKD-EPI: Chronic Kidney Disease Epidemiology Collaboration
CNS: Central nervous system
DDAVP: Desmopressin
DH: Dehydration-induced hypernatraemia
eGFR: Estimated glomerular filtration rate
ICU: Intensive care unit
NDI: Nephrogenic diabetes insipidus
NSAR: Non-steroidal anti-rheumatic
SAPS II: Simplified Acute Physiology Score II
SO: Salt overload
SSRI: Selective serotonin reuptake inhibitor
