# Supplementary figures and images for: Copeptin levels and commonly used laboratory parameters in hospitalised patients with severe hypernatraemia - the “Co-MED study”
Source: Crit Care. 2018 Feb 9;22:33. doi: 10.1186/s13054-018-1955-7 (PMC5806470; doi:10.1186/s13054-018-1955-7)

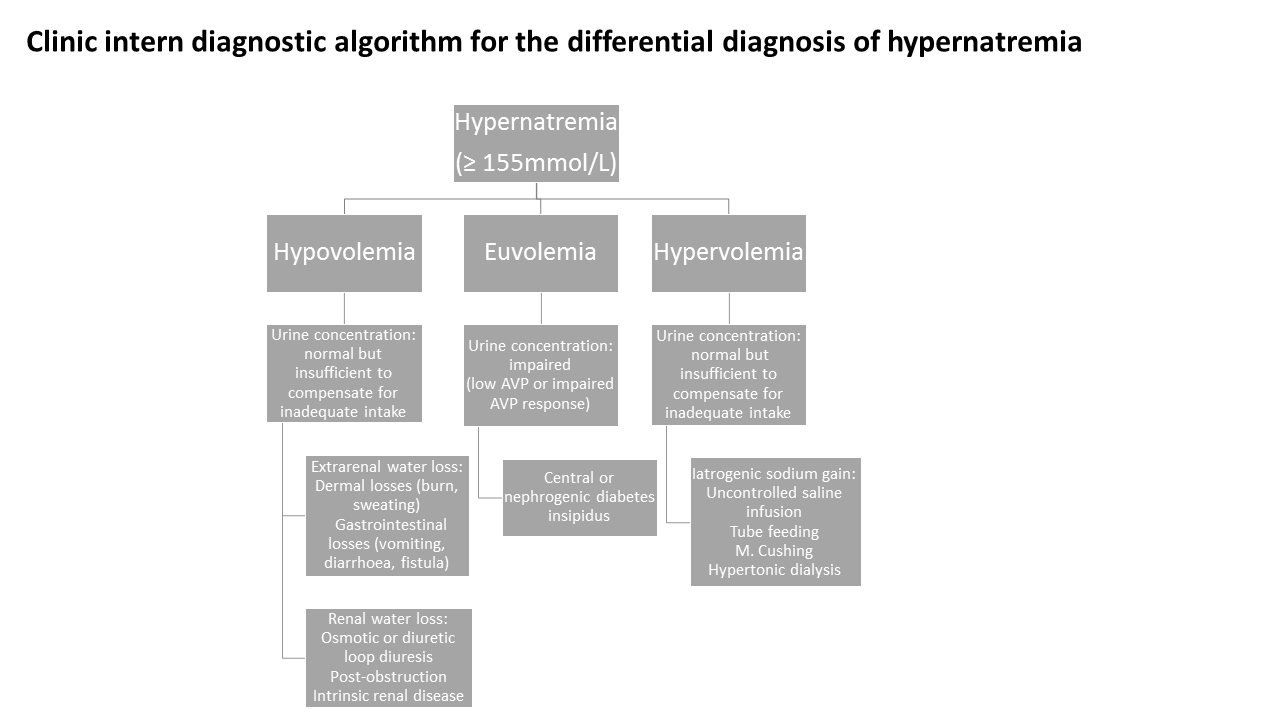

Supplement: Supplementary file 1 — Clinical diagnostic algorithm for the differential diagnosis of hypernatremia. (TIFF 143 kb) [file 13054_2018_1955_MOESM1_ESM.tiff]
